# Supplementary material for: Lp-PLA2 silencing ameliorates inflammation and autophagy in nonalcoholic steatohepatitis through inhibiting the JAK2/STAT3 pathway
Source: PeerJ. 2023 Jun 26;11:e15639. doi: 10.7717/peerj.15639 (PMC10309053; doi:10.7717/peerj.15639)

HE

NCD

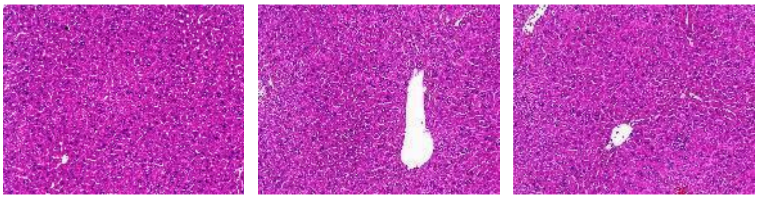

HFD

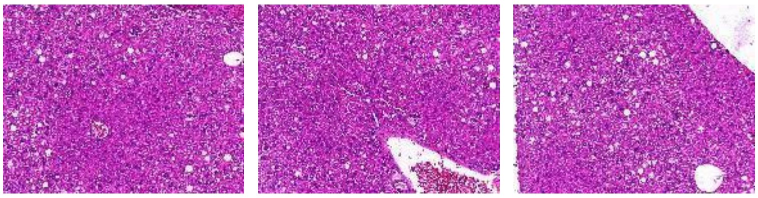

Sh-NC+HFD

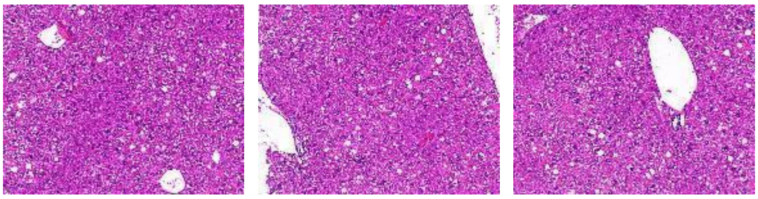

Sh-Lp-PLA2 +HFD

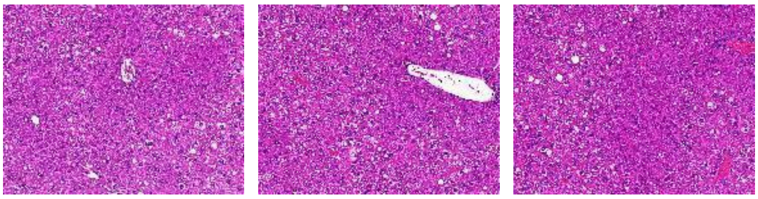

Rapamycin+HFD

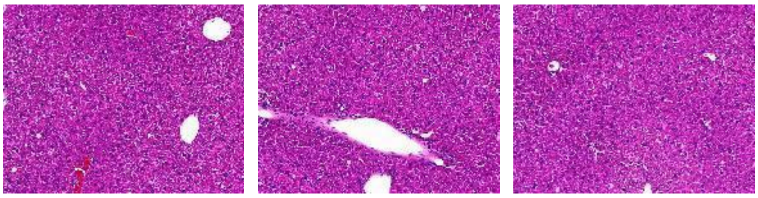

Sh-Lp-PLA2+ Rapamycin+HFD

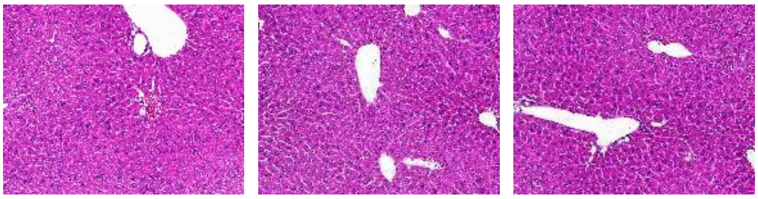

Oli Red O

NCD

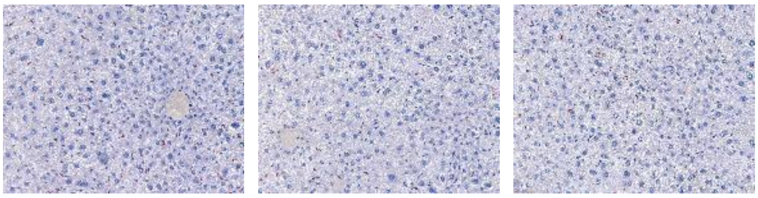

HFD

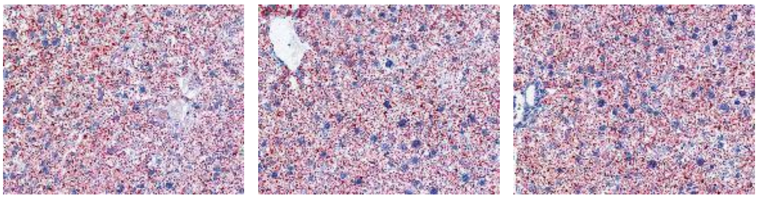

Sh-NC+HFD

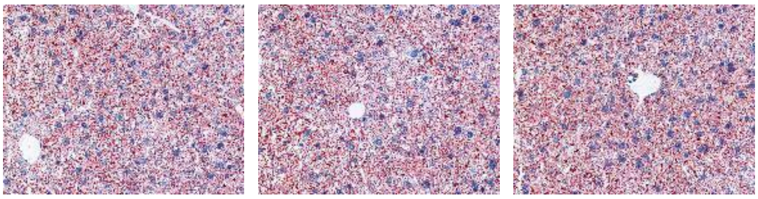

Sh-Lp-PLA2 +HFD

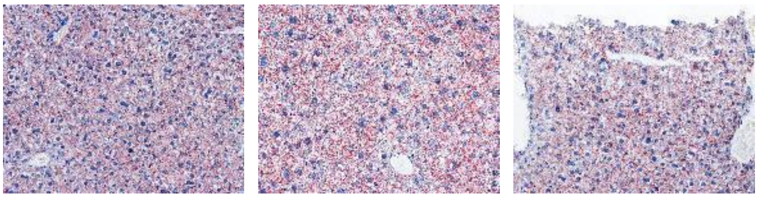

Rapamycin+HFD

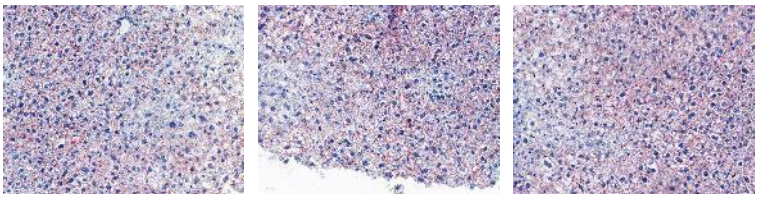

Sh-Lp-PLA2+ Rapamycin+HFD

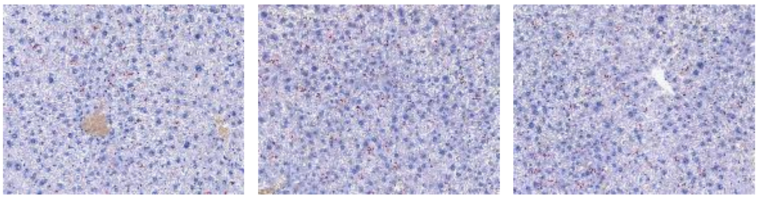

MASSON

NCD

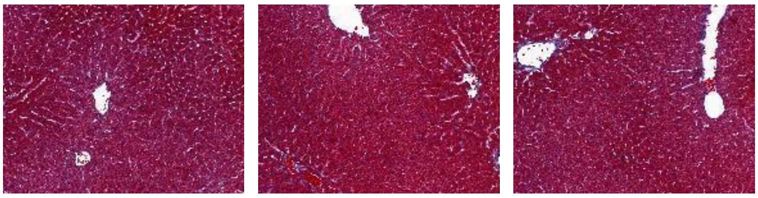

HFD

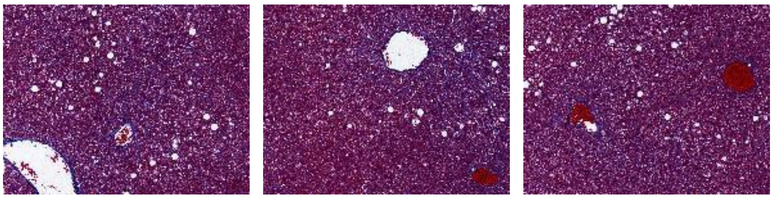

Sh-NC+HFD

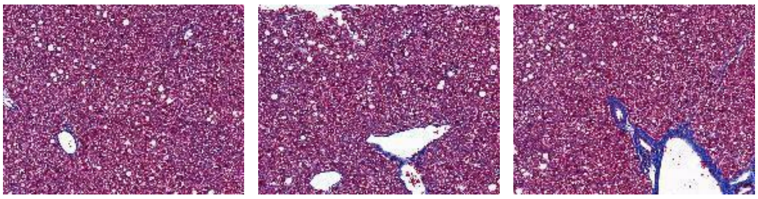

Sh-Lp-PLA2 +HFD

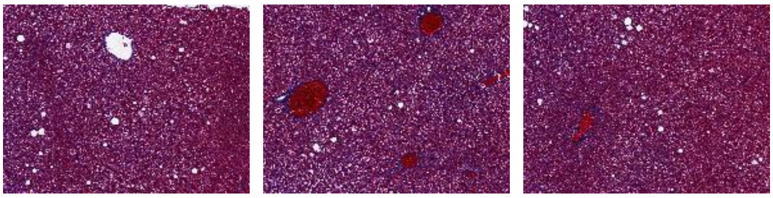

Rapamycin+HFD

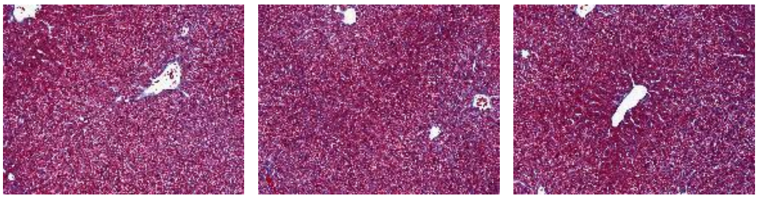

Sh-Lp-PLA2+ Rapamycin+HFD

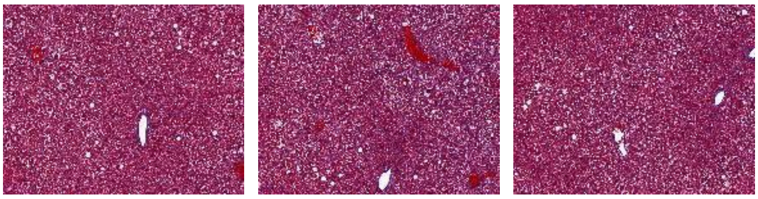

Supplement: Supplemental Information 1 [file peerj-11-15639-s001.zip › raw data/Figure 2.pdf]
