# Supplementary material for: Lp-PLA2 silencing ameliorates inflammation and autophagy in nonalcoholic steatohepatitis through inhibiting the JAK2/STAT3 pathway
Source: PeerJ. 2023 Jun 26;11:e15639. doi: 10.7717/peerj.15639 (PMC10309053; doi:10.7717/peerj.15639)
Supplement: Supplemental Information 1 [file peerj-11-15639-s001.zip › raw data/Figure 4.pdf]

Normal

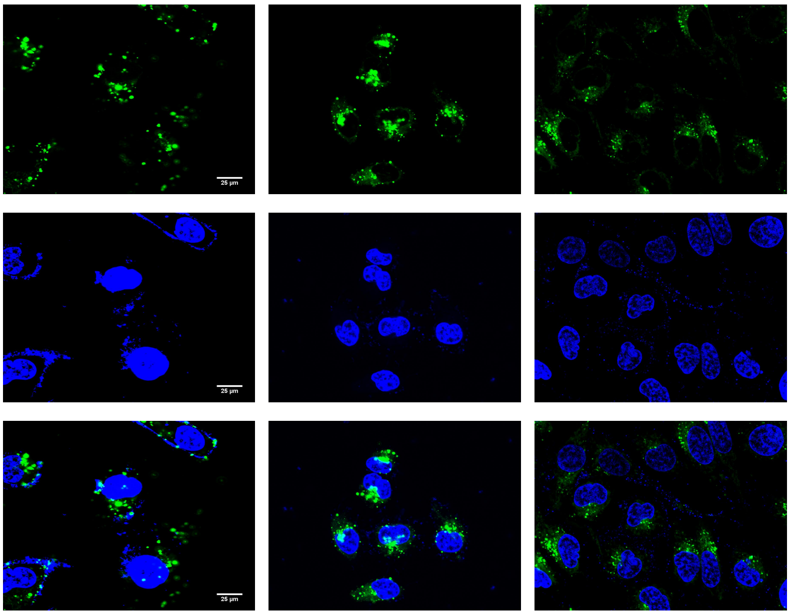

NASH

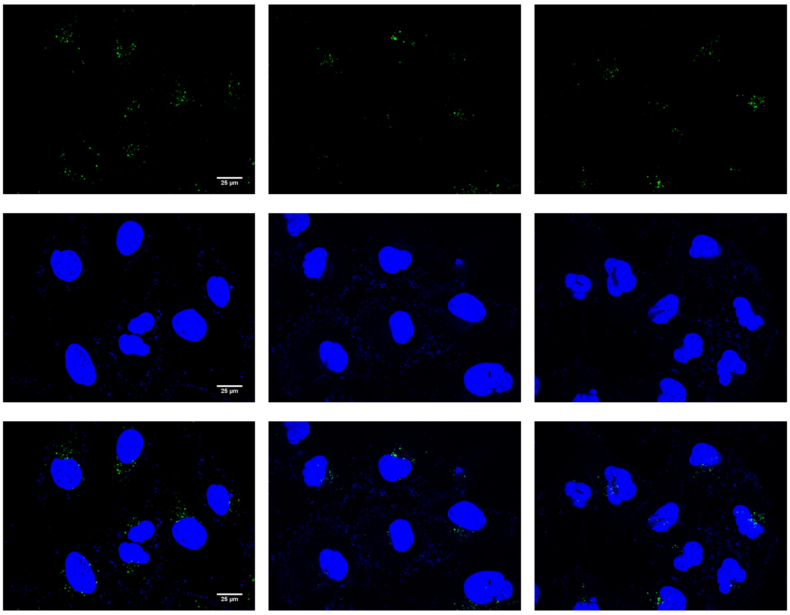

Sh-NC+NASH

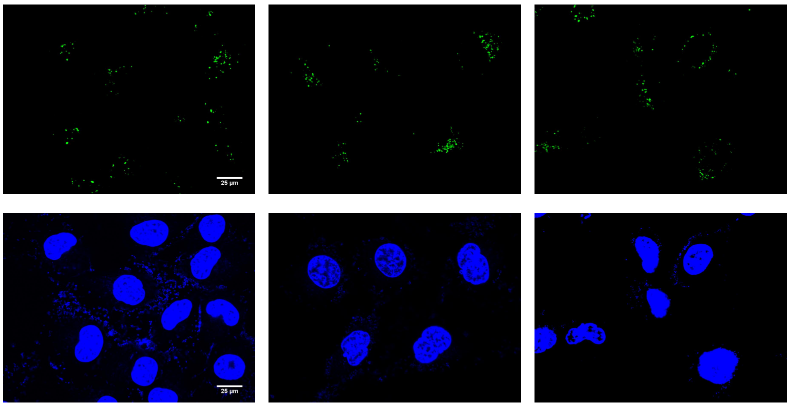

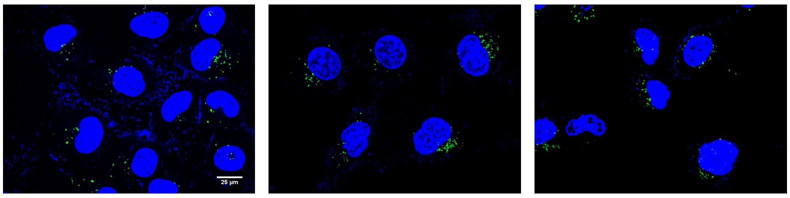

Sh-Lp-PLA2+NASH

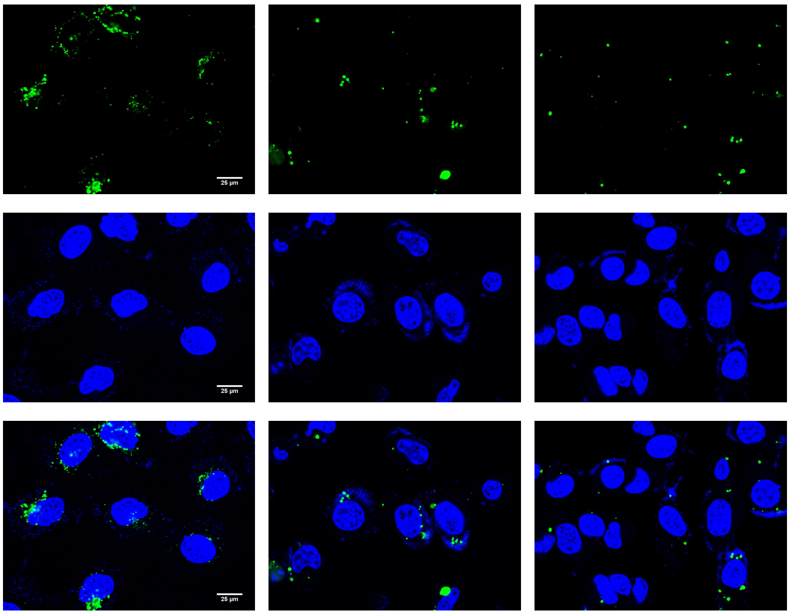

Rapamycin+NASH

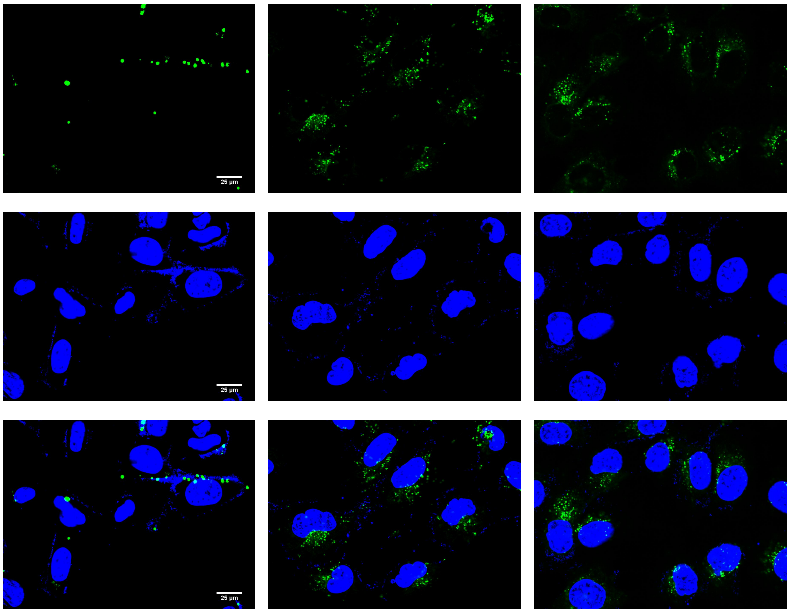

Sh-Lp-PLA2+ Rapamycin+NASH

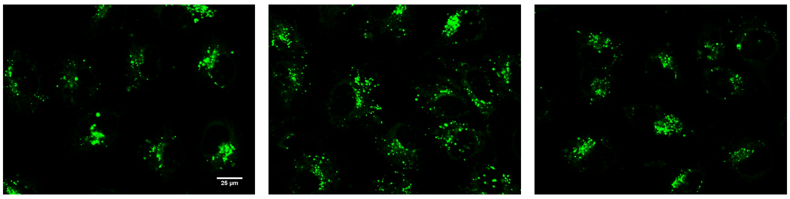

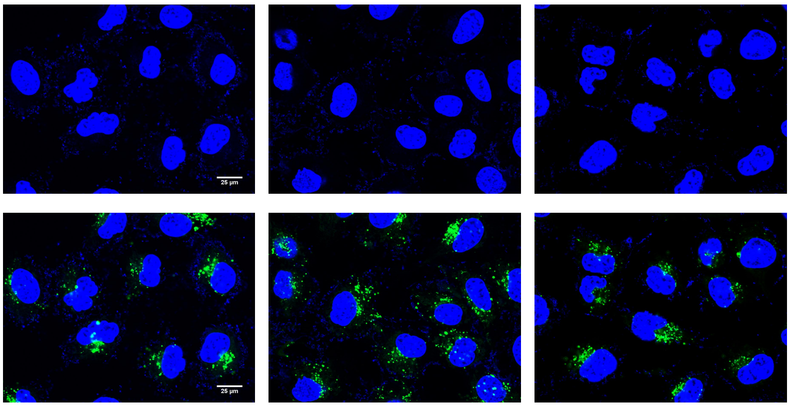

JAK2-inhibitor+NASH

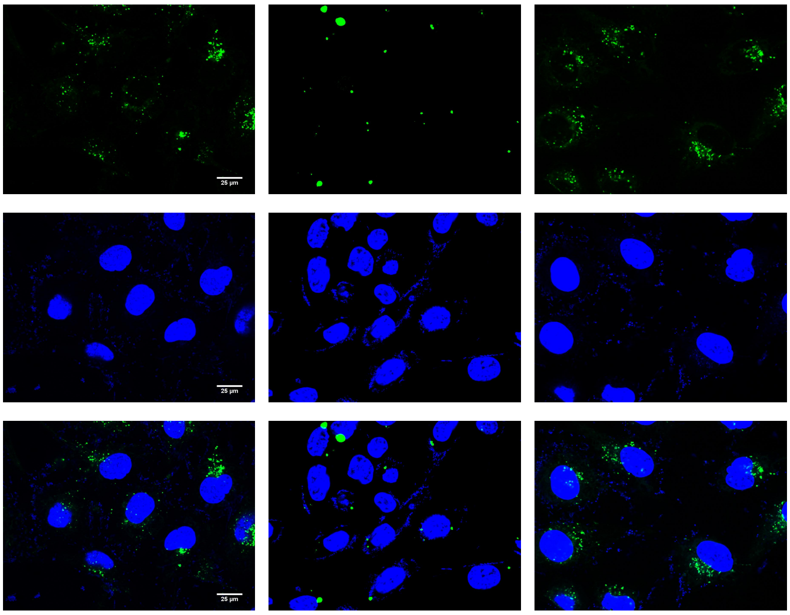

Sh-Lp-PLA2+ JAK2-inhibitor+NASH

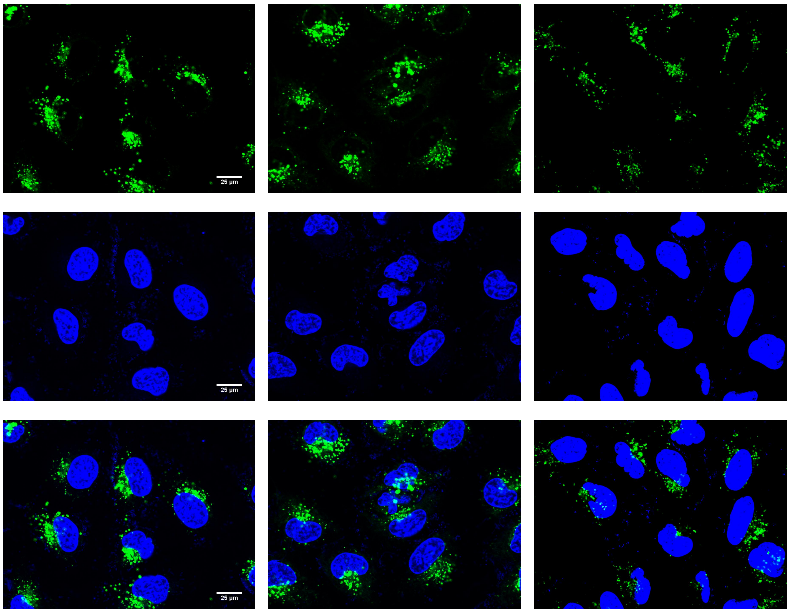

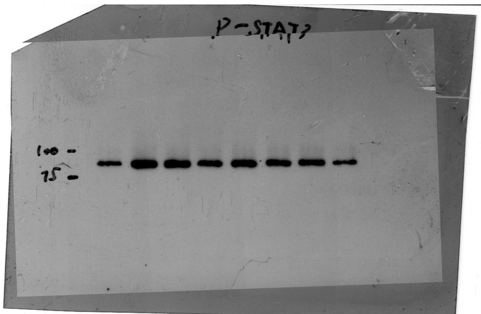
P-STAT3
100
75

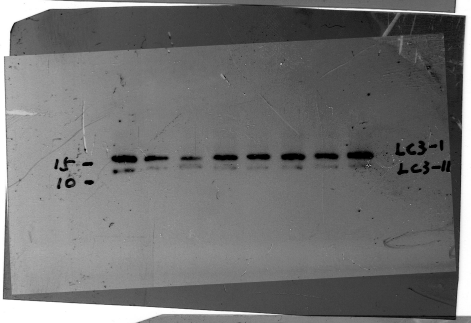
15
10
LC3-I
LC3-II

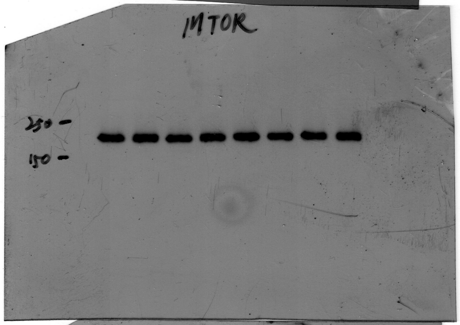
MTOR
250
150

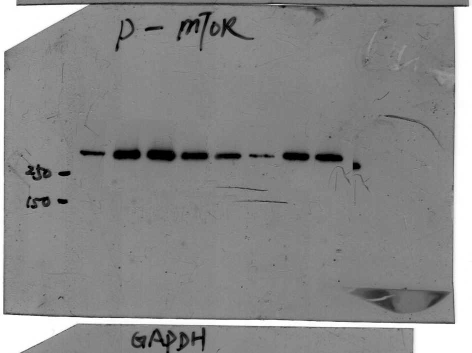
P-MTOR
250
150

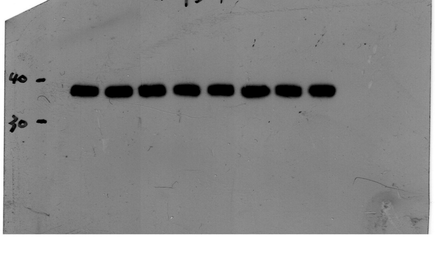
GAPDH
40
30

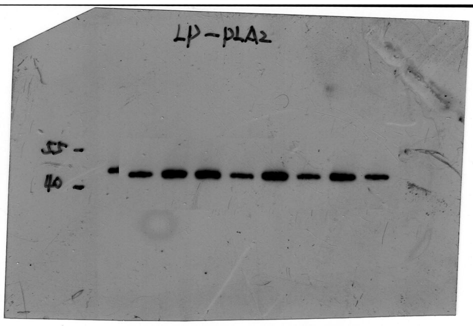
LP-PLA2
55
40

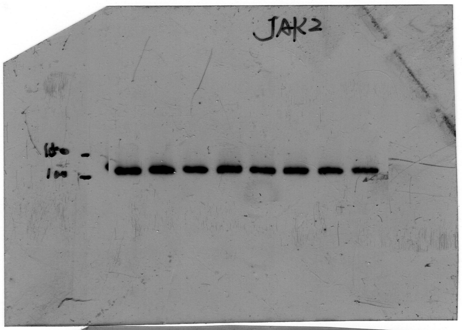
JAK2

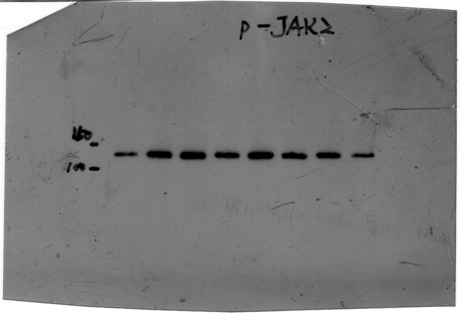
P-JAK2

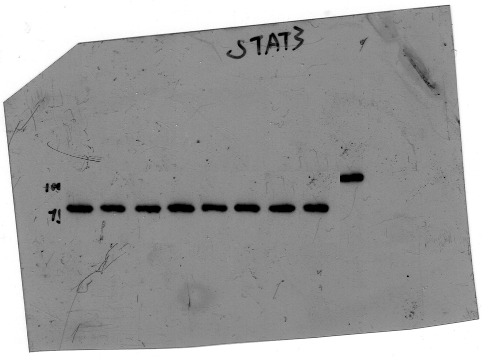
STAT3
