# Supplementary material for: Lp-PLA2 silencing ameliorates inflammation and autophagy in nonalcoholic steatohepatitis through inhibiting the JAK2/STAT3 pathway
Source: PeerJ. 2023 Jun 26;11:e15639. doi: 10.7717/peerj.15639 (PMC10309053; doi:10.7717/peerj.15639)

Normal

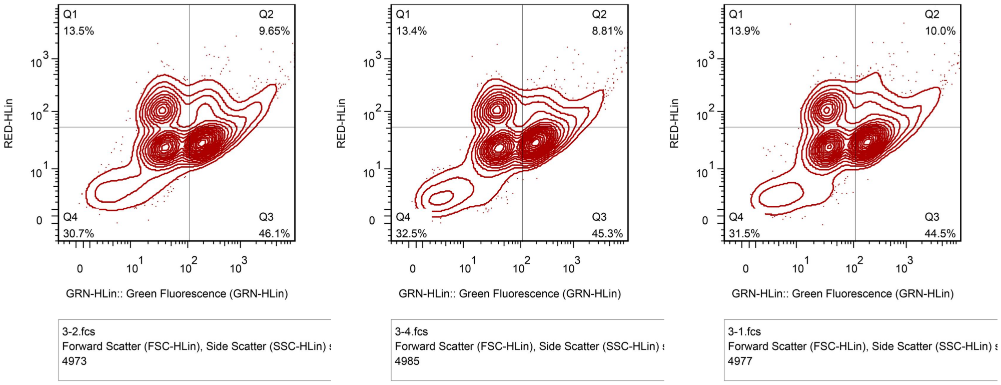


NASH

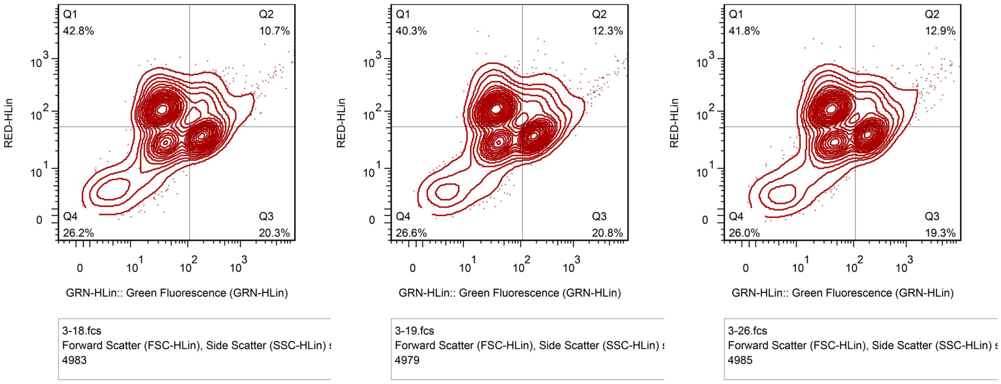


Sh-NC +NASH

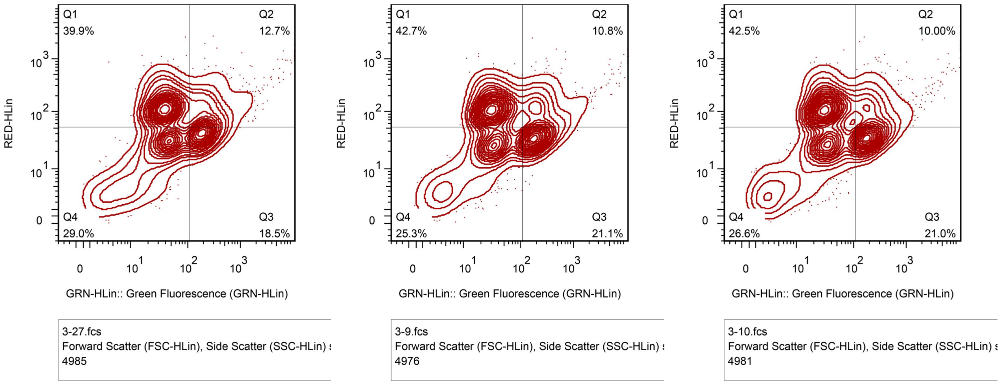


Sh-Lp-PLA2 +NASH

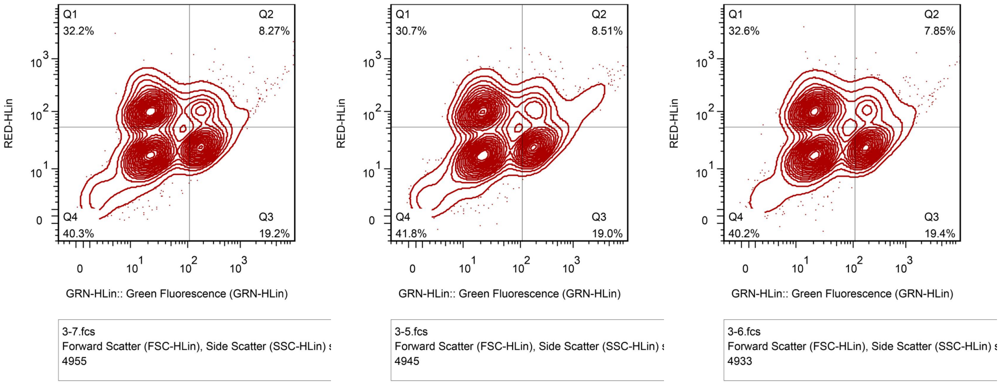


Rapamycin+NASH

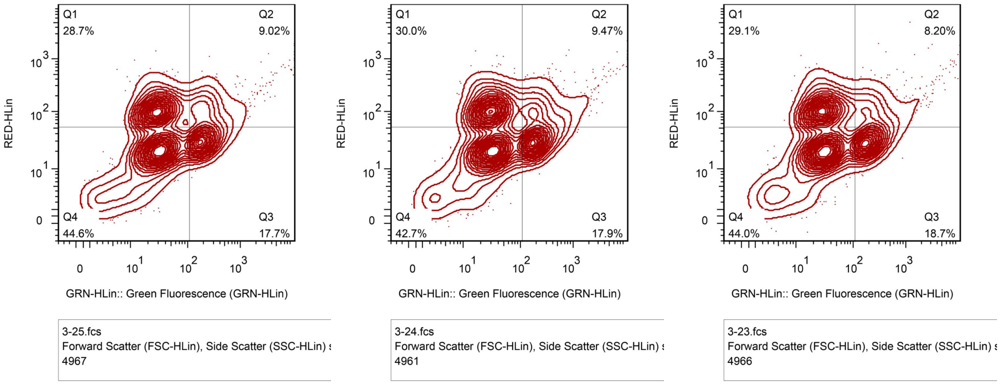


Sh-Lp-PLA2+ Rapamycin+NASH

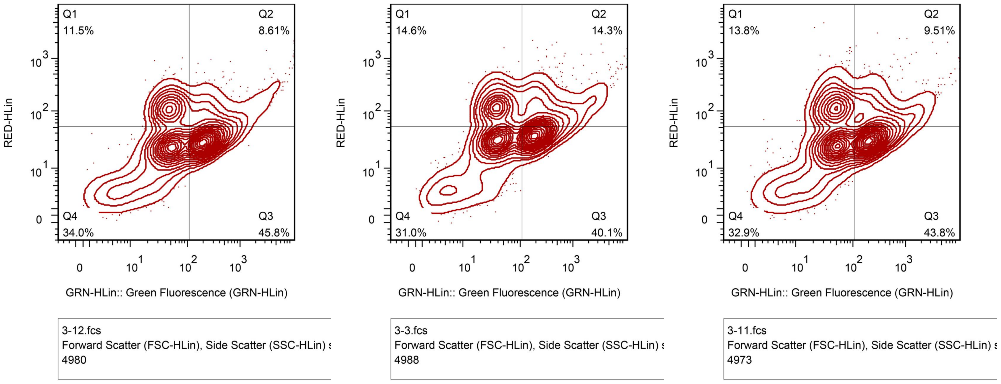


JAK2-inhibitor+NASH

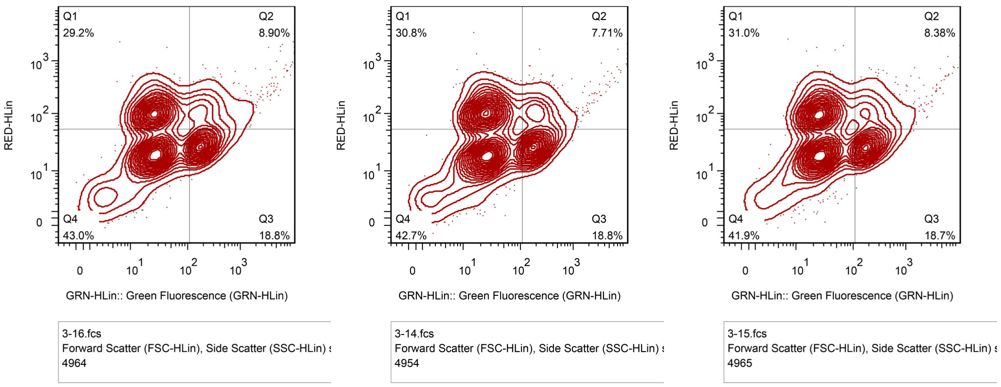


Sh-Lp-PLA2+JAK2-inhibitor+NASH

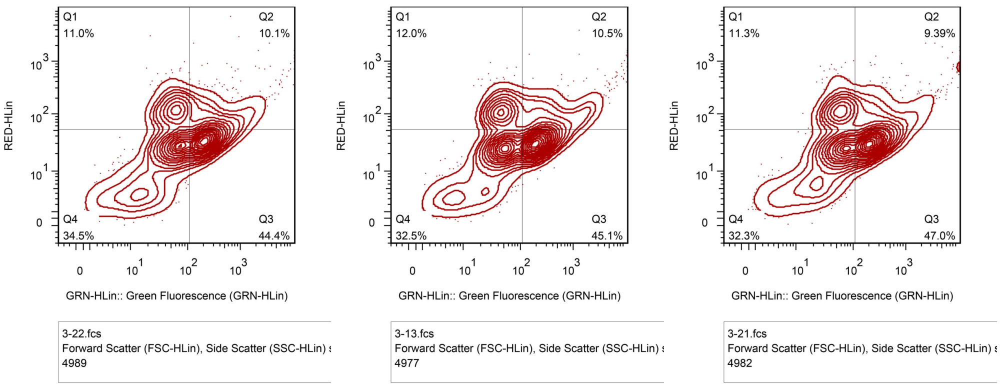

Supplement: Supplemental Information 1 [file peerj-11-15639-s001.zip › raw data/Figure 5.pdf]
