# Supplementary figures and images for: Lp-PLA2 silencing ameliorates inflammation and autophagy in nonalcoholic steatohepatitis through inhibiting the JAK2/STAT3 pathway
Source: PeerJ. 2023 Jun 26;11:e15639. doi: 10.7717/peerj.15639 (PMC10309053; doi:10.7717/peerj.15639)

NCD

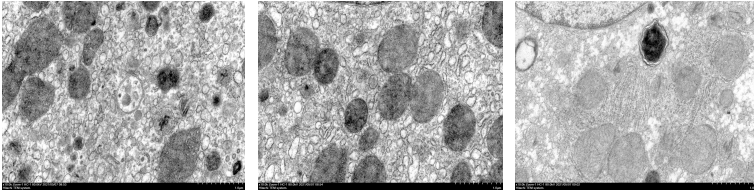

HFD

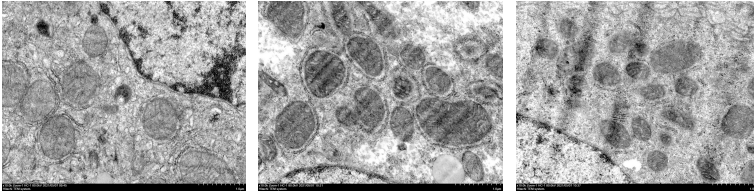

Sh-NC+HFD

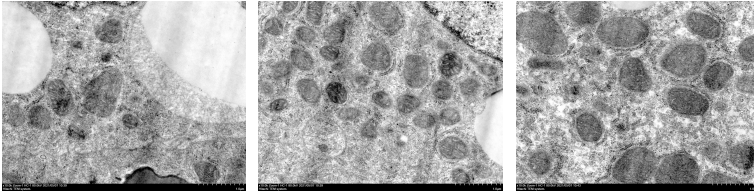

Sh-Lp-PLA2 +HFD

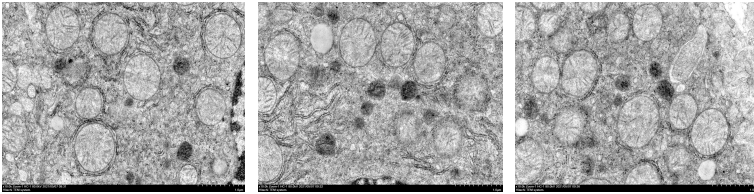

Rapamycin+HFD

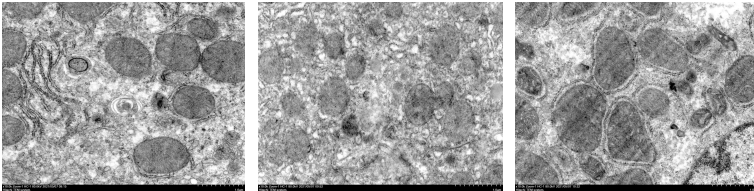

Sh-Lp-PLA2+ Rapamycin+HFD

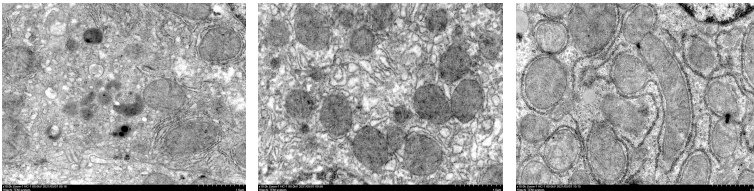

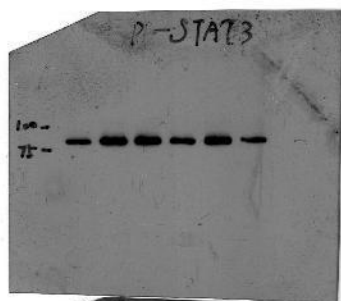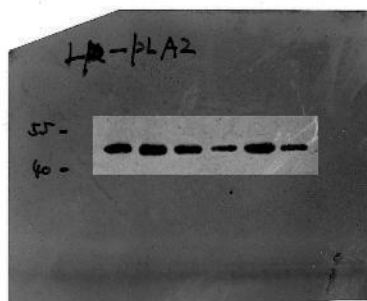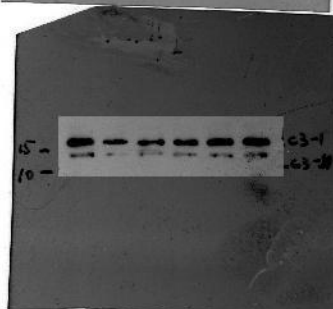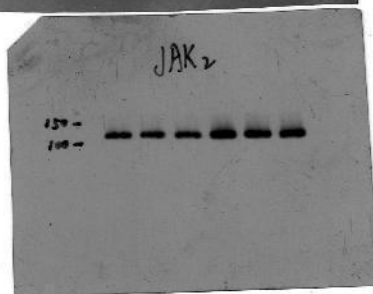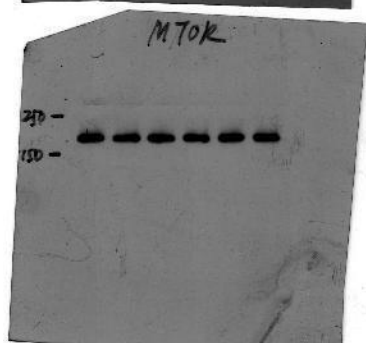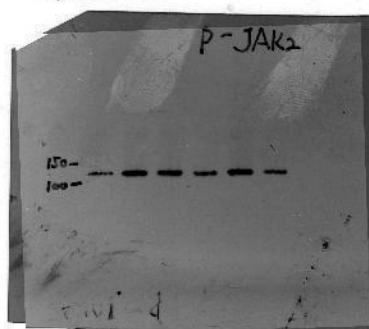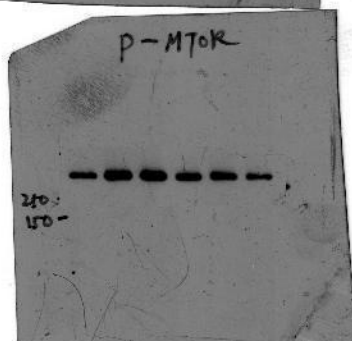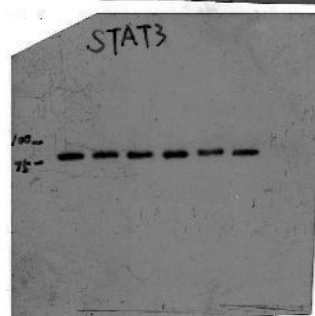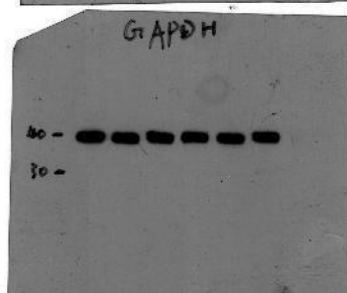

Supplement: Supplemental Information 1 [file peerj-11-15639-s001.zip › raw data/Figure 3.pdf]

Figure 3B

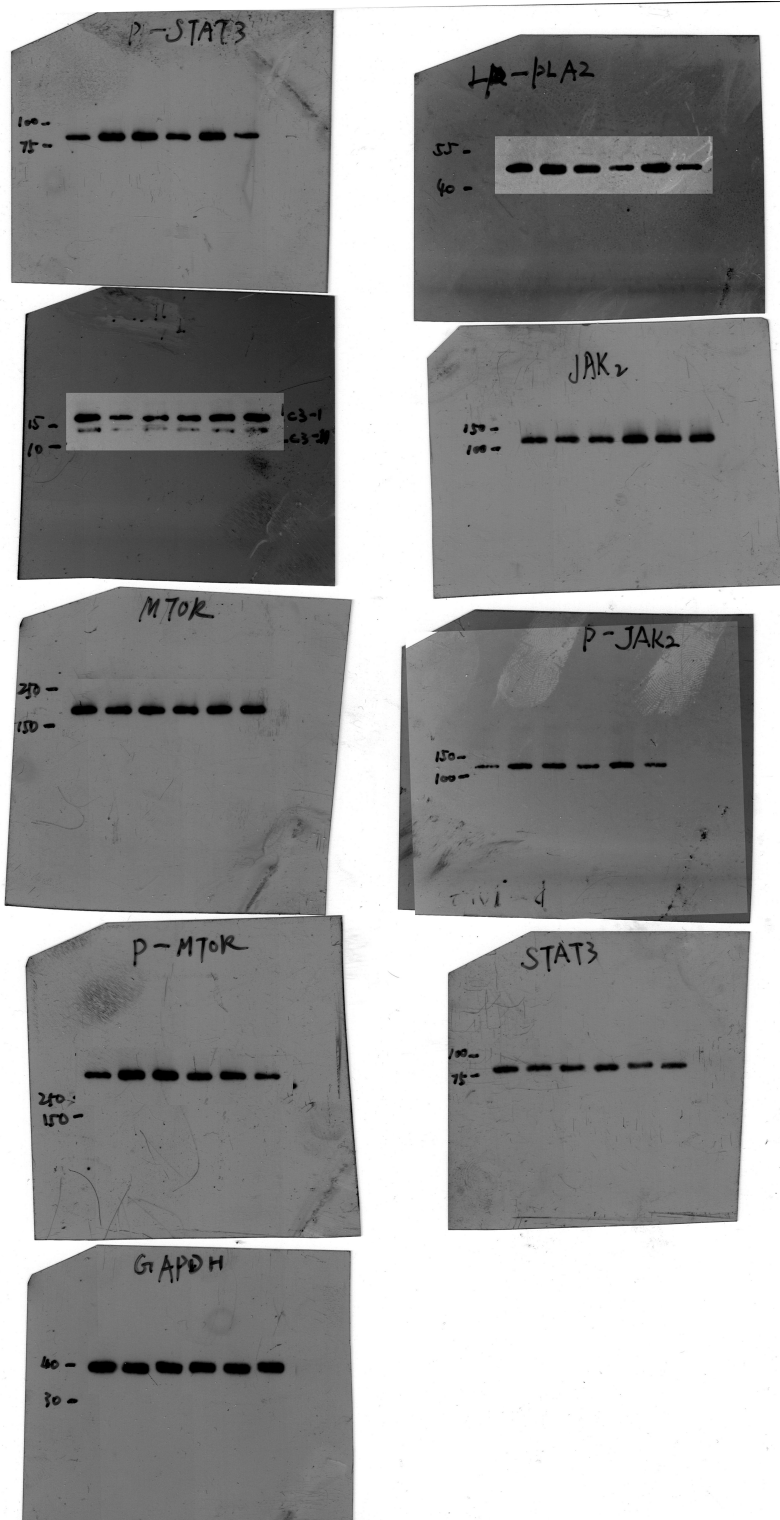

Figure 4B

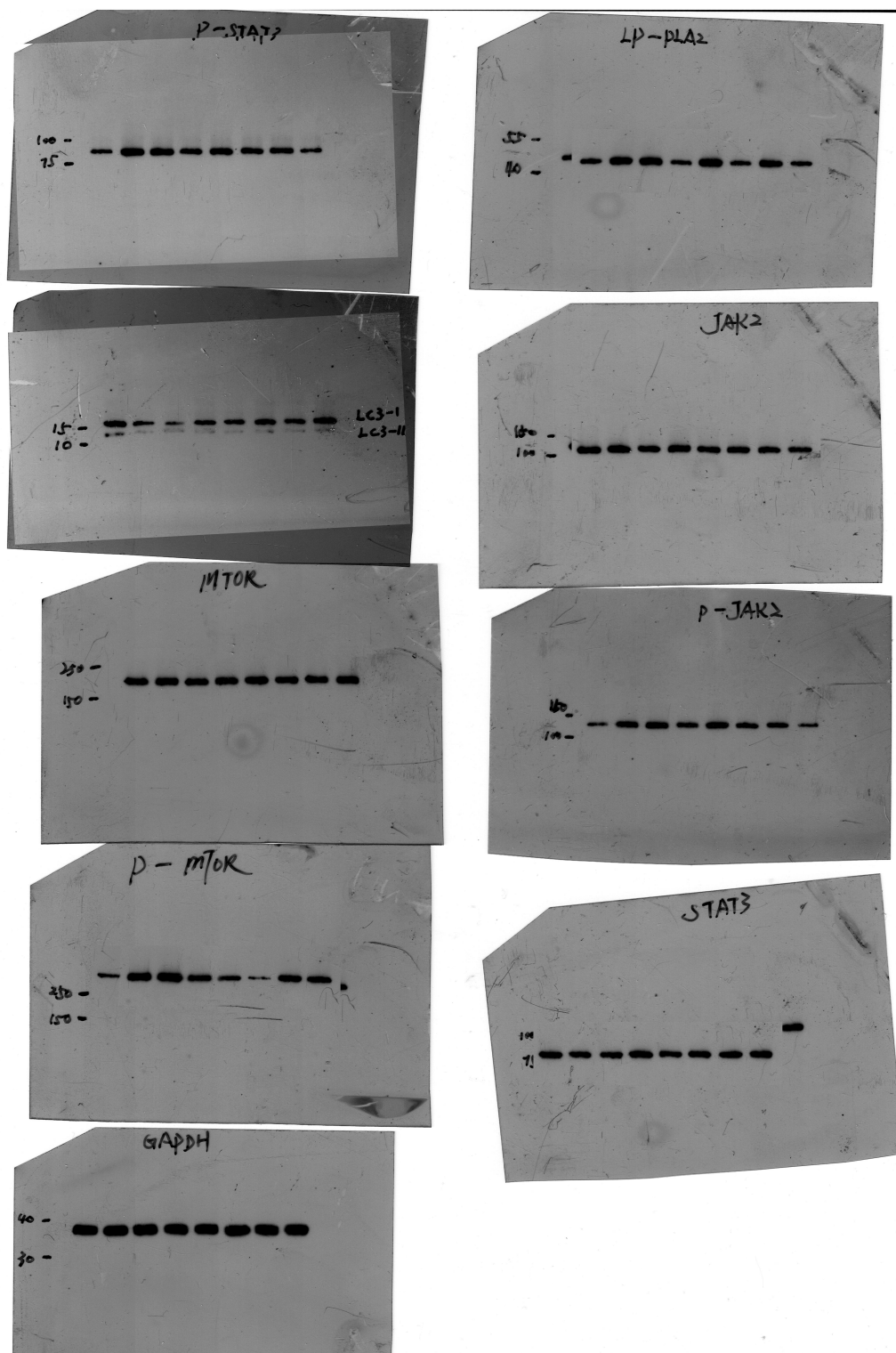

Supplement: Supplemental Information 3 [file peerj-11-15639-s003.pdf]
